# Altered mitochondrial lymphocyte in overweight schizophrenia patients treated with atypical antipsychotics and its association with cognitive function

**DOI:** 10.3389/fimmu.2023.1325495

**Published:** 2024-01-03

**Authors:** Yaoyao Zhang, Wei Tang, Bei Tang, Kaili Fan, Ke Zhao, Xinyu Fang, Hui Lin

**Affiliations:** ^1^ Department of Psychiatry, The Affiliated Kangning Hospital of Wenzhou Medical University Zhejiang Provincial Clinical Research Center for Mental Disorder, Wenzhou, Zhejiang, China; ^2^ Department of Education, Children’s Hospital of Nanjing Medical University, Nanjing, Jiangsu, China; ^3^ Department of Psychiatry, Wenzhou Seventh People’s Hospital, Wenzhou, Zhejiang, China; ^4^ Department of Psychiatry, Wenzhou Medical University, Wenzhou, Zhejiang, China; ^5^ Department of Psychiatry, the Affiliated Brain Hospital of Nanjing Medical University, Nanjing, Jiangsu, China; ^6^ Department of Psychiatry, The Second People`s Hospital of YuHuan, Taizhou, Zhejiang, China

**Keywords:** schizophrenia, atypical antipsychotic medications, mitochondrial lymphocyte, overweight, cognition

## Abstract

**Objective:**

Increasing evidence indicated that schizophrenia and obesity are associated with altered mitochondrial and immune function. In this study, we investigated the levels of CRP (C-reactive protein) and mitochondrial lymphocytes in chronically treated schizophrenia patients with atypical antipsychotic medications and further explored the relationship between mitochondrial lymphocyte and weight gain as well as cognitive function in these patients.

**Methods:**

We evaluated the mitochondrial lymphocyte count of 97 patients (53 overweight, 44 non-overweight) and 100 healthy controls using mitochondrial fluorescence staining and flow cytometry (NovoCyte, Agilent Technologies, US). The serum CRP was measured by high-sensitivity enzyme-linked immunosorbent assay (ELISA). Clinical symptoms and cognitive function of the patients were assessed using the Positive and Negative Syndrome Scale (PANSS) and the Repeatable Battery for the Assessment of Neuropsychological Status (RBANS).

**Results:**

The results showed that mitochondrial lymphocyte counts of CD3+ T, CD3+CD4+ T, and CD3+CD8+ T cells in schizophrenia patients were higher than in the control group (p < 0.05). Additionally, overweight patients had significantly higher mitochondrial lymphocyte counts of CD3+ T and CD3+CD4+ T cells compared to schizophrenia patients with normal weight. Stratified analysis by gender revealed that there was a statistically significant difference in CD3+CD4+ mitochondrial lymphocyte count in male patients (p = 0.014) and a marginal trend toward significance in female patients (p = 0.058). Furthermore, the mitochondrial lymphocyte counts of CD3+ T and CD3+CD4+ T cells, as well as CRP levels, were positively correlated with BMI in schizophrenia patients, but the mitochondrial lymphocyte counts of CD3+CD4+ T cells were negatively correlated with the language scale in the RBANS.

**Conclusion:**

Our study results provide evidence for the association between altered mitochondrial T lymphocyte and weight gain as well as cognitive impairment in schizophrenia patients treated with atypical antipsychotic medications.

## 1 Introduction

Schizophrenia is a profoundly debilitating mental disorder that affects approximately 1% of the global population, imposing significant burdens on both individuals and society (1). In addition to its primary symptoms, growing evidence suggests a higher prevalence of obesity among individuals with schizophrenia compared to the general population (2–4). This phenomenon is primarily attributed to the use of antipsychotics, notably atypical antipsychotics. Existing studies have revealed that the utilization of atypical antipsychotics in patients with schizophrenia is associated with a substantial incidence of obesity, ranging from 40% to 60% (5). Nonetheless, the precise mechanisms that underlie the relationship between schizophrenia and antipsychotic-induced obesity remain only partially understood.

As we know, obesity is characterized by a chronic state of low-grade inflammation (6, 7), which involves immune system dysregulation. This inflammation stems from adipose tissue dysfunction, potentially contributing to the lymphocyte mitochondrial dysfunction and subsequent systemic low-grade inflammation. Furthermore, the disruption of the insulin signaling pathway, often observed in individuals with obesity, can impair mitochondrial function, leading to increased oxidative stress. Additionally, the altered lipid metabolism in obese individuals may give rise to excessive production of reactive oxygen species, consequently causing damage to the mitochondria (8). Substantial evidence demonstrates that impaired mitochondrial function in patients with schizophrenia is associated with the activation of immune inflammation through the release of reactive oxygen species (ROS) and damage-associated molecular markers (9). On the one hand, disturbances like oxidative stress can lead to erroneous folding of the mitochondrial permeability transition (MPT) pore, resulting in non-selective permeability of the inner mitochondrial membrane, membrane potential collapse, mitochondrial swelling, calcium overload, and cytochrome c release, thus triggering caspase activation and apoptosis (10). On the other hand, the mitochondrial damage promotes the release of danger-associated molecules (DAMPs) and ROS, which play a critical role in activating and sustaining inflammatory immune responses, while the inflammatory response can further exacerbate mitochondrial damage (11). Taken together, the change of mitochondrial lymphocyte count may reflect the damage of mitochondria to some extent.

Previous studies have found a connection between metabolic syndrome induced by antipsychotic medication and immune inflammation (12). Mitochondria, the energy-producing centers within cells, are crucial for normal cellular functioning. Some studies have indicated impaired lymphocyte mitochondrial function in patients receiving treatment with atypical antipsychotic medications(13). Furthermore, the metabolic syndrome induced by medication may contribute to lymphocyte mitochondrial damage (8). Dysregulation of immune inflammation can lead to oxidative stress and mitochondrial dysfunction, thereby accelerating mitochondrial damage. In this context, abnormal lymphocyte mitochondrial function may represent a potential mechanism for weight gain and related metabolic abnormalities. Interestingly, some previous literature reported that schizophrenia patients with obesity had more severe cognitive impairment than those without obesity, and the relationship between cognitive impairment and immune inflammation in schizophrenia has also been extensively reported. For instance, our research indicates that immune dysregulation induced by atypical antipsychotic medications can cause a decrease in BDNF, further contributing to cognitive decline (12). Increased inflammation has been repeatedly associated with cognitive deficits, as evidenced in the majority of studies (14, 15). More intriguingly, clinical studies have revealed that short-term use of antipsychotics can effectively improve cognitive function in patients with schizophrenia, but long-term use of atypical antipsychotics leads to weight-gain-related metabolic disorders that exacerbate cognitive impairment in patients, a phenomenon that may be influenced by immune-inflammatory activation (12, 16). However, other studies have found no statistical difference in cognitive impairment between patients with schizophrenia and those without obesity. Since limited studies have focused on this topic, more research should be conducted.

In this study, we used specific fluorescent probes bound to living mitochondria and specific antibodies to CD3, CD4, CD8, and CD19 to label mitochondria and their lymphocyte subsets (17). Then the number of lymphocyte subsets was detected by flow cytometry to reflect the degree of mitochondrial damage and immune inflammation status of individuals. We here aim to investigate whether the weight gain induced by atypical antipsychotics exacerbates cognitive impairment in patients with schizophrenia and whether immune inflammation related to mitochondrial lymphocyte plays a role in the weight gain and cognitive impairment induced by atypical antipsychotics in schizophrenia.

## 2 Methods

### 2.1 Participants

From January 2022 to December 2022, we recruited 97 patients with schizophrenia and 100 healthy controls at the Affiliated Kangning Hospital of Wenzhou Medical University. All participants signed informed consent forms. The clinical trial protocol has been approved by the Ethics Committee of Kangning Hospital in Wenzhou. This study was performed in strict accordance with the Declaration of Helsinki and all other relevant national and international regulations.

All patients met the following criteria: (1) age between 18 and 65 years; (2) a diagnosis of schizophrenia according to the Diagnostic and Statistical Manual of Mental Disorders Fifth Edition (DSM-5); (3) treated with atypical antipsychotics (such as clozapine, quetiapine, olanzapine, ziprasidone, aripiprazole, risperidone, amisulpride, paliperidone) at least six months; (4) with a normal-weight before the antipsychotic drugs used through the retrospective report of patients and their legal guardians. Patients with the following conditions were excluded from the study: (1) diagnosed with other psychiatric disorders according to DSM-V; (2) had autoimmune diseases, heart diseases, hepatobiliary and gastrointestinal diseases, blood diseases, or neurological diseases; (3) pregnancy or lactation; (4) using laxatives, prebiotics, or anti-inflammatory drugs within a week before the study; (5) had taken antibiotics or probiotics in the past three months. Healthy controls with no personal or family history of psychiatric disorders were recruited from the Wenzhou region via advertisement, the inclusion criteria for the control group as follows: (1) without current or previous history of psychiatric disorders; (2) age between 18 and 65 years;(3)with normal BMI. Exclusion criteria are the same as those for the schizophrenia group. Patients were divided into overweight and non-overweight groups based on the criteria of BMI (Body Mass Index) ≥ 24 kg/m^2 or waist circumference ≥ 85 cm for females, and ≥ 90 cm for males (18).

### 2.2 Measures

All the recruited patients underwent a demographic interview, a systematic assessment of medical history, and an interview based on the Structured Clinical Interview for DSM-IV Axis I Disorders. The severity of psychotic symptoms was evaluated using the Positive and Negative Syndrome Scale (PANSS) (19). It consists of 30 items scored from 1 to 7, including three subscales (PANSS positive subscale, PANSS negative subscale, PANSS general psychopathology subscale), with higher scores indicating greater symptom burden. In addition, five factors of PANSS also considered in the current study (20). The cognitive factor is composed of three PANSS items: “Conceptual disintegration” (P2), “Difficulty in abstract thinking” (N5), and “Poor attention” (G11), which is used to measure cognitive function. The depressive factor consists of three other PANSS items: (G2), (G3), and (G6). The positive symptom factor consists of P1, P3, P6, and G9. The excited-hostile symptom factor consists of P4, P7, G8, and G14. The negative symptom factor consists of N1, N2, N3, N4, N6, and G7.

The Repeatable Battery for the Assessment of Neuropsychological Status (RBANS) was used to assess cognitive function in patients. The 12-item RBANS consists of five subsets, corresponding to the following five neuropsychological processes: immediate memory, visuospatial function, language, attention, and delayed memory (21). The RBANS has good validity and reliability in Chinese people and is suitable for the cognitive evaluation of patients with schizophrenia (22). Generally, a higher RBANS score reflects a better cognitive function.

Whole blood samples were collected from all fasting participants into sterile EDTA anticoagulant tubes (5 ml) and BD Vacutainer serum-separating tubes (5 ml) (Becton, Dickinson, and Company; Franklin Lakes, New Jersey, United States). EDTA anticoagulant tubes used for mitochondrial lymphocyte detection requires whole blood specimens stored at 4°C in a refrigerator until analysis according to the manufacturer’s instructions (NovoCyte, Agilent Technologies, US). Cells were labeled with a 96-well plate containing a mitochondria-specific dye (UB1024, UBBiotechnology Co., Ltd., Hangzhou, China) that can be activated by a 633-nm laser. The plate was wrapped with aluminum foil to keep it from light; the content was incubated at room temperature for 3–5 minutes and centrifuged for 1 minute at 250×g. Then, immunophenotypic antibodies were used to prepare the “mixed reagent” to label cell populations, including CD3-FITC (UB104411, UBBiotechnology Co., Ltd., Hangzhou, China), CD4-PE-Cy7 (UB105441), CD8-PE (UB106421), and CD45-PerCP-Cy5.5 (UB109481). Contents in the plate were added with 20 µl of “mixed reagent” and incubated for 15 minutes. Then, 100 µl of anticoagulated human peripheral blood was added and incubated for 15 minutes at room temperature, away from light. Hemolysin (NH Lysis Solution, 10×) 400 µl was processed similarly. Finally, flow cytometry (NovoCyte, Agilent Technologies, US) was used to detect the counts of T lymphocyte subsets (CD3, CD4, and CD8) in the peripheral blood.

The BD Vacutainer serum-separating tubes used for CRP measurement were inverted and left to clot at room temperature for 30 minutes. After clotting, the tube was centrifuged at 2000× g for 10 minutes at 4°C to separate the red blood cells from the serum. The tube was centrifuged at 2000× g for 10 min at 4°C to separate red blood cells from serum, and the serum was collected and stored at -80°C. CRP was measured from serum by high-sensitivity enzyme-linked immunosorbent assay (ELISA) according to the manufacturer’s instructions (IBL-international, Hamburg, Germany) (23, 24). Ten microliters of well-mixed serum from each patient were serially diluted to 1:1000 with sample diluent. A five-point standard curve of 0, 0.4, 1, 5, and 10 μg/ml CRP from the manufacturer, diluted to the same concentration as the samples, was used to calibrate and quantify concentrations. Absorbance was read at 450 nm using a BMG Optima Plate Reader. Forty-seven values were below the minimum detectable concentration of approximately 0.02 μg/ml, and were, therefore, recorded as 0.02 μg/ml. Sample concentrations ranged from 0.02 to 11.94 μg/ml.

### 2.3 Statistical analysis

Statistical analysis were performed using SPSS software version 26.0 (SPSS, 146 Chicago, IL). The independent samples t-test, the analysis of variance (ANOVA) or the chi-square test was used to compare the demographic, clinical, and blood differences between groups as appropriate. We further used the analysis of covariance (ANCOVA) to control for the effects of confounding variables such a sex, age, medication duration, DAD (Daily antipsychotic medication dosage, chlorpromazine equivalents) and age of onset. In addition, the gender stratification analysis was also conducted. Then, the Spearman-Rho correlation was conducted to assess the potential relationship between the level of mitochondrial lymphocyte count in schizophrenia and BMI, the severity of clinical symptoms (reflected by PANSS scores) as well as cognitive function. Least Significant Difference (LSD) correction was applied to each test to adjust for multiple testing. For all the tests, P ≤ 0.05 (two-tailed) was considered statistically significant.

## 3 Results

### 3.1 Differences in demographic and mitochondrial lymphocyte count between schizophrenia patients treated with atypical antipsychotics and healthy controls

The demographic and clinical characteristics of the patients and controls are presented in **Table 1**. There were no statistically significant differences in age and gender between the schizophrenia group and the control group (p > 0.05). Compared to the control group, schizophrenia patients had a higher level of mitochondrial lymphocyte count in CD3+ T, CD3+CD4+ T, and CD3+CD8+ T lymphocytes. The mitochondrial lymphocyte count indices of CD3+ (t = 3.535, p = 0.001), CD3+CD4+ (t = 2.953, p = 0.004), and CD3+CD8+ (t = 3.498, p = 0.001) T lymphocytes in the schizophrenia group were lower compared to the control group. There were no significant differences in mitochondrial lymphocyte count of CD4+/CD8+, CD3+CD4+ (%), and CD3+CD8+ (%) between schizophrenia patients and healthy controls (all p > 0.05).

**Table 1:** Differences in Demographic and Mitochondrial Lymphocyte Count in Lymphocytes Between Patients and Controls.

	schizophrenia (N = 97)	Healthy controls (N = 100)	t/X^2^	p
			t	p
Age (year)	39.88 ± 9.828	40.23 ± 6.82	0.293	0.770
Mito-CD4+/CD8+	1.70 ± 0.69	1.66 ± 0.72	-0.392	0.696
Mito-CD3+CD4+	683.63 ± 291.91	843.13 ± 451.59	2.953	0.004^**^
Mito-CD3+CD4+ (%)	47.70 ± 77.31	37.92 ± 7.14	-1.260	0.209
Mito-CD3+	1213.39 ± 434.46	1519.00 ± 743.50	3.535	0.001^**^
Mito-CD3+ (%)	85.13 ± 133.94	68.84 ± 7.93	-1.214	0.226
Mito-CD3+CD8+	429.84 ± 191.52	557.25 ± 307.96	3.498	0.001^**^
Mito-CD3+CD8+ (%)	26.36 ± 7.46	25.51 ± 7.57	-0.801	0.424
			X^2^	p
Sex (male/female)	51/46	61/39	1.424	0.252

Mito, Mitochondria.

**p<0.01. Data were presented in Mean ± SD.

### 3.2 Differences in terms of demographic characteristics, mitochondrial lymphocyte count, and clinical features between overweight and normal weight groups treated with atypical antipsychotics

**Figure 1:**
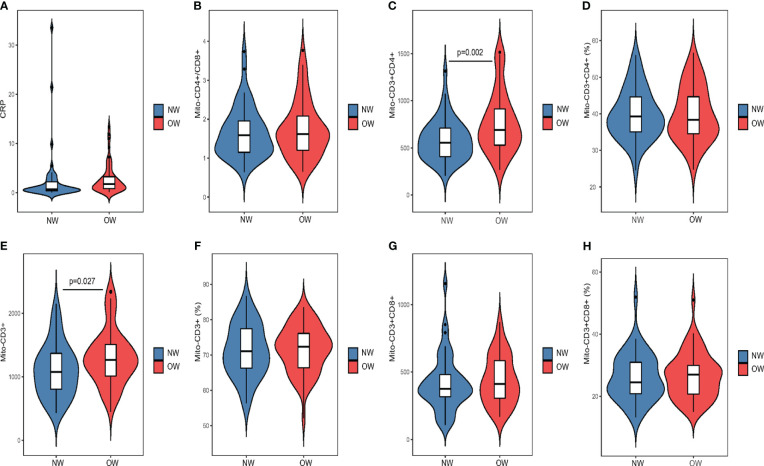
Comparison of lymphocyte mitochondrial parameters between overweight and normal weight groups using atypical antipsychotic drugs **(A)** Comparsion of CRP between groups. **(B)** Comparsion of Mito-CD4+/CD8+ between groups. **(C)** Comparsion of Mito-CD3+CD4+ between groups. **(D)** Comparsion of Mito-CD3+CD4+(%) between groups. **(E)** Comparsion of Mito-CD3+ between groups. **(F)** Comparsion of Mito-CD3+(%) between groups. **(G)**Comparsion of Mito-CD3+CD8+ between groups. **(H)** Comparsion of Mito-CD3+CD8+(%) between groups. NW, normal-weight patients; OW, overweight patients.

There were no significant differences in demographic factors (gender, age, age of onset, education level), cognitive function, and PANSS scores (total score and subscale scores) between the overweight patients and normal-weight patients (all p > 0.05). Our results showed that the levels of mitochondrial lymphocyte count in T cells, including CD3+ (t = -2.245, p = 0.027) and CD3+CD4+ (t = -3.258, p = 0.002), were considerably higher in the overweight patients compared to the normal-weight patients (**Figure 1**). After controlling for confounding factors such as medication duration, age, sex, DAD and age of onset, there was still a statistically significant difference between the two groups in terms of mitochondrial CD3+CD4+ T lymphocytes (F = 2.605, p = 0.023). We further conducted the gender stratification analysis, the CD3+CD4+ mitochondrial lymphocyte count showed a statistically significant difference (F = 2.605, p = 0.023) in male patients, and a marginal trend toward significance (p = 0.058) in female patients (see in **Table S1** in **Supplementary Materials**). However, there were no significant differences in mitochondrial lymphocyte count of other lymphocytes and CRP (C-reactive protein) levels between these two patient groups (**Table 2**). Additionally, we further compared the mitochondrial lymphocyte count among overweight patients, normal weight patients and healthy controls, the results are showed in **Table S2** (see in **Supplementary Materials**), the difference in CD3+CD4+ T lymphocytes (LSD corrected p = 0.021), but not CD3+ (LSD corrected p = 0.118), survived after LSD correction.

**Table 2:** Comparisons between schizophrenic patients with and without overweight.

	overweight (N = 53)	Non-overweight (N = 44)	t/X^2^	p
Age (year)	40.30 ± 10.18	39.36 ± 9.49	-0.466	0.642
Sex (male/female)	27/26	24/20	0.125	0.724
Education (years)	7.69 ± 3.37	8.43 ± 2.94	1.131	0.261
Age of onset (years)	26.13 ± 11.42	25.02 ± 8.62	-0.531	0.597
Duration of medication (years)	12.92 ± 7.62	13.98 ± 8.52	0.642	0.523
DAD(mg)	409.89 ± 252.97	425.65 ± 296.55	0.282	0.778
CRP	2.79 ± 3.07	2.59 ± 5.93	-0.216	0.830
Mito-CD4+/CD8+	1.74 ± 0.73	1.66 ± 0.65	-0.581	0.562
Mito-CD3+CD4+	764.96 ± 320.74	585.66 ± 218.78	-3.258	0.002^**^
Mito-CD3+CD4+ (%)	39.83 ± 7.53	39.84 ± 6.86	0.010	0.992
Mito-CD3+	1301.79 ± 431.55	1106.91 ± 418.30	-2.245	0.027^*^
Mito-CD3+ (%)	71.63 ± 6.56	71.20 ± 7.42	-0.303	0.763
Mito-CD3+CD8+	441.05 ± 180.10	416.34 ± 205.73	-0.631	0.530
Mito-CD3+CD8+ (%)	26.55 ± 7.37	26.13 ± 7.64	-0.276	0.783
RBANS				
Immediate memory	52.85 ± 13.25	53.14 ± 14.61	0.101	0.919
Attention	75.77 ± 15.19	74.70 ± 15.09	-0.346	0.730
Visuospatial	73.74 ± 15.03	75.57 ± 15.48	0.590	0.557
Delayed memory	61.08 ± 16.99	61.75 ± 18.89	0.185	0.854
Language	68.30 ± 14.29	69.41 ± 13.70	0.387	0.700
Total score	59.15 ± 10.84	60.73 ± 11.44	0.695	0.489
PANSS				
Total score	104.77 ± 11.71	107.32 ± 15.34	0.926	0.357
Cognitive factor	12.30 ± 2.02	12.61 ± 2.46	0.685	0.495
Depressive factor	8.66 ± 2.30	9.02 ± 2.43	0.754	0.452
Positive symptoms	23.21 ± 3.33	23.75 ± 4.57	0.675	0.501
Negative symptoms	14.40 ± 3.59	15.30 ± 4.33	1.119	0.266
Excitation	12.02 ± 3.39	11.66 ± 3.19	-0.534	0.594

DAD, Daily antipsychotic medication dosage (chlorpromazine equivalents); CRP, C-reactive protein; Mito, Mitochondria; RBANS, the Repeatable Battery for the Assessment of Neuropsychological Status; SCWT, the Stroop Color-Word Test.

*p<0.05, **p<0.01. Data were presented in Mean ± SD.

### 3.3 Associations between the severity of symptoms, cognition, and the degree of mitochondrial lymphocyte

**Figure 2:**
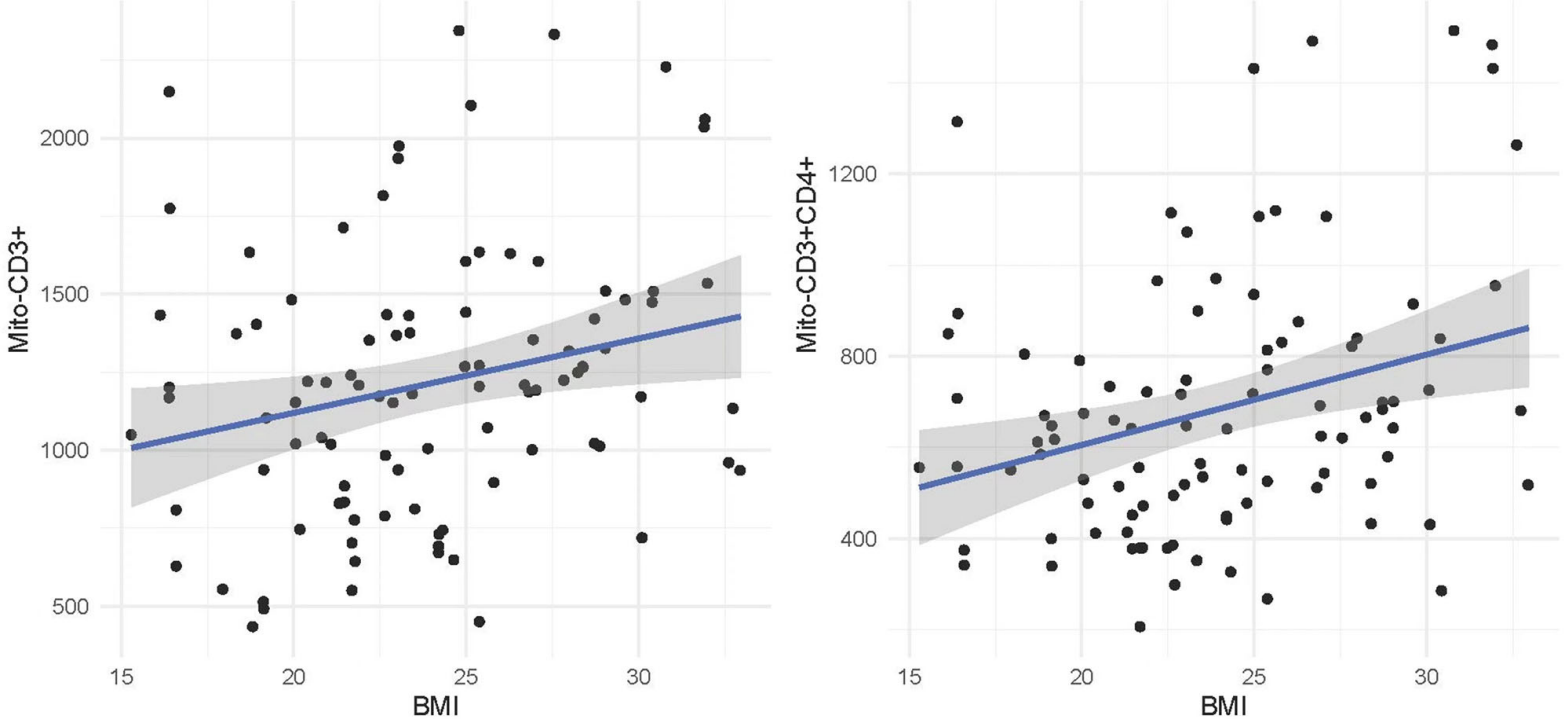
The correlation between mitochondrial counts of CD3+ and CD3+CD4+ lymphocytes and BMI in schizophrenia patients using atypical antipsychotic drugs. Mito, Mitochondria; BMI, Body Mass Index.

Spearman’s correlation was used to analyze the correlation between the PANSS score, BRANS score, CRP, and the level of mitochondrial lymphocyte count, as the data were not normally distributed. No correlation between the degree of mitochondrial lymphocyte count was found with the total, negative, and general score of PANSS. In addition, no significant association was found between the age of onset or education and the mitochondrial lymphocyte count (all P > 0.05). However, the result showed that the increase in CRP (r = 0.261, p = 0.01), CD3+ (r = 0.254, p = 0.012), and CD3+CD4+ (r = 0.270, p = 0.007) (**Table 3**; **Figure 2**) lymphocyte mitochondrial lymphocyte count is positively correlated with the increase in BMI. The CD4+/CD8+ mitochondrial ratio is positively correlated with age (r = 0.218, p = 0.032) and the severity of positive psychotic symptoms (r = 0.258, p = 0.011). The CD3+CD4+ mitochondrial lymphocyte count is negatively correlated with language scores in the BRANS (r = -0.230, p = 0.024).

**Table 3:** Correlations Between Clinical Features and Mitochondrial Damage in Lymphocytes.

Clinical symptoms	Age		BMI		Language subscore		Positive subscore	
	r	p	r	p	r	p	r	p
CRP	-0.175	0.086	0.261	0.010^*^	-0.004	0.971	0.040	0.701
Mito-CD3+	-0.031	0.761	0.254	0.012^*^	-0.085	0.406	0.048	0.640
Mito-CD3+CD4+	0.034	0.741	0.270	0.007^**^	-0.230	0.024^*^	0.019	0.850
Mito-CD4+/CD8+	0.218	0.032^*^	0.024	0.817	-0.127	0.214	0.258	0.011^*^

CRP, C-reactive protein; Mito, Mitochondria.

*p<0.05, **p<0.01.

## 4 Discussion

This study compared and analyzed cognitive function and mitochondrial lymphocyte-related parameters between patients with schizophrenia treated with atypical antipsychotics and healthy controls, as well as between patients with schizophrenia who were overweight and those with normal weight. Additionally, it further explored the relationship between weight gain induced by atypical antipsychotic medications and mitochondrial lymphocyte count, as well as its impact on cognitive function. Our findings revealed the following: 1) Patients with schizophrenia exhibited a lower count of mitochondrial lymphocytes in peripheral T lymphocytes compared to the healthy control group. 2) Overweight patients showed significantly higher mitochondrial levels in CD3+ T and CD3+CD4+ T lymphocytes in comparison to non-overweight patients. 3) The CD4+/CD8+ T lymphocyte mitochondrial ratio displayed a positive correlation with both age and the severity of positive symptoms. 4)A negative correlation was observed between CD3+CD4+ mitochondrial T lymphocyte count and language scores in RBANS.

This study found significant differences in the count of mitochondrial lymphocytes in peripheral blood T cells between individuals with schizophrenia and healthy controls, consistent with earlier research findings. Prior studies have consistently demonstrated the association of mitochondrial dysfunction with various psychiatric disorders (9), including schizophrenia (25, 26), bipolar disorder (27), and depression (28). Furthermore, it has been observed that individuals with mitochondrial dysfunction are more susceptible to experiencing psychiatric symptoms (29, 30). Genetic abnormalities linked to energy metabolism and oxidative stress may serve as underlying factors contributing to mitochondrial dysfunction in schizophrenia. Research has unveiled reduced expression of genes associated with oxidative phosphorylation in pyramidal neurons (31), along with defects in genes such as lactate dehydrogenase A, NADH dehydrogenase, and ATP synthase. These genes are specific to schizophrenia and are not found in depression or bipolar disorder (32, 33). Remarkably, current research has not detected mitochondrial damage in B lymphocytes, a phenomenon potentially related to the involvement of cellular immunity rather than humoral immunity in the pathological processes of schizophrenia.

The study also observed an increase in lymphocyte mitochondrial count in the overweight group due to atypical antipsychotic medications, although current research on this topic is limited. Considering the presence of various gender differences in schizophrenia (34, 35), we further conducted gender stratification analysis. We found that the difference in mitochondrial CD3+CD4+ counts between male overweight and normal weight schizophrenia patients was more pronounced compared to female patients. This suggests that future investigations in this area should also consider gender as an important factor. This could be attributed to the fact that obese patients often experience chronic low-grade inflammation (36), and the inflammatory response is associated with an increase in mitochondrial count. In an inflammatory state, immune cells are activated and release inflammatory cytokines and mediators, potentially leading to lymphocyte proliferation and an increase in mitochondrial count. Furthermore, obese patients require more energy to meet the demands of their tissues and organs due to weight gain. Mitochondria are the primary energy-producing organelles, and the increase in mitochondrial count may serve to meet the heightened energy demands of obese patients (37). Studies have indicated that certain medications containing antioxidant components, such as N-acetylcysteine and coenzyme Q10, can improve mitochondrial activity in peripheral blood immune cells (38). Additionally, some antipsychotic medications, like Olanzapine and Chlorpromazine, may protect mitochondrial function through their anti-inflammatory and antioxidant effects or by regulating mitochondrial gene expression (39, 40). However, certain typical antipsychotic drugs can impair mitochondrial function by reducing the activity of mitochondrial complex I, decreasing ATP production, and dissipating mitochondrial membrane potential (Psychiatric drugs impact mitochondrial function in the brain and other tissues (38). The study also found a positive correlation between weight gain induced by antipsychotic medication and treatment response (41). Therefore, monitoring patient weight in the clinical management of schizophrenia may be beneficial for treatment and prognosis.

Furthermore, our investigation revealed an association between the severity of schizophrenia symptoms and the T lymphocyte mitochondrial CD4+/CD8+ ratio. However, research findings regarding this relationship are inconsistent. While some studies have reported an increase in the CD4+/CD8+ ratio in specific individuals with schizophrenia (42), there remains ongoing debate on this topic. Some studies suggest that the elevated CD4+/CD8+ ratio may be linked to negative symptoms in schizophrenia (such as attention deficits and emotional blunting) (43), with a weaker association observed with positive symptoms (such as hallucinations and delusions), which contrasts with our research findings. Conversely, other studies have not identified any correlation between this ratio and specific symptom types (13). Similarly, research findings regarding the relationship between age and the T lymphocyte mitochondrial CD4+/CD8+ ratio are also subject to controversy. In theory, during normal immune aging, the number and function of CD4+ T cells might decline, while the number of CD8+ T cells could remain stable or slightly increase, resulting in a lower CD4+/CD8+ ratio. However, certain studies have indicated that with increasing age, the T lymphocyte mitochondrial CD4+/CD8+ ratio may increase (44). This could be attributed to age-related changes in the immune system, heightened inflammatory responses, and declining mitochondrial function. In 2016, a study identified a significant increase in the T lymphocyte mitochondrial CD4+/CD8+ ratio among elderly individuals, which demonstrated a positive correlation with age (45). Nevertheless, other studies have not observed significant age-related changes or have even identified a contrary trend (46). These discrepancies may be attributed to differences in study design, sample characteristics, and methodologies employed. In summary, the relationship between the T lymphocyte mitochondrial CD4+/CD8+ ratio and the severity of schizophrenia symptoms, as well as its association with age, remains inconsistent and necessitates further research. Conducting more in-depth and comprehensive studies is crucial to gain a better understanding of the complex associations between these factors. Such research will be instrumental in unraveling the pathophysiological mechanisms of schizophrenia and may offer valuable insights into potential therapeutic strategies.

A limited amount of research has explored the association between CD3+CD4+ T lymphocyte mitochondria and language impairment. The mitochondrial function of CD3+CD4+ lymphocytes in schizophrenia is significantly impaired, possibly due to persistent low-level inflammation. Improving mitochondrial function may enhance synaptic plasticity and alleviate cognitive dysfunction (47). However, in a study involving 18 individuals with chronic schizophrenia, more severe negative symptoms and cognitive deficits were associated with a relative decrease in the number of dendritic cells, regulatory T cells, and memory T cells, which contradicts our findings (48).

## 5 Limitations

We should take into consideration several limitations of this study. Firstly, it is essential to note that this is a cross-sectional study and cannot establish a causal relationship between mitochondrial lymphocyte count and cognitive function in schizophrenia. It cannot reflect long-term changes and dynamic processes. Future studies with longitudinal design are needed to observe the dynamic changes in mitochondrial markers under the use of AAPs. Secondly, given the possible differences in the effects of different antipsychotics on the immune system (49), which may have an influence on our results, however, the small sample size of this study prevented us from performing a subgroup analysis by antipsychotic types. Future studies are warranted to subdivide the groups of patients depending on the treatment received in order to demonstrate that the effect on the mitochondria is independent of the type of AAP administrated. Thirdly, although we recruited patients with a normal weight before the use of antipsychotic drugs through retrospective reports from patients and their legal guardians, there may be some recall bias. Previous studies have found a high incidence of obesity in treatment-naïve patients as well. Therefore, future research should include treatment-naïve patients (both obese and non-obese) and healthy controls (overweight or normal weight) to exclude the potential impact of the disease itself on mitochondrial function, among other factors, and utilize a cohort design to investigate this issue. Fourthly, our study did not include an assessment of cognitive function in a control group; thus, we were unable to explore the relationship between T lymphocyte mitochondrial function and cognition in healthy individuals. Despite these limitations, our preliminary study holds significant value as it provides initial data on the occurrence and treatment of schizophrenia. As mitochondrial parameters cannot currently be used as standalone biomarkers for diagnosis, further research incorporating *in vivo* quantitative neuroimaging, gene expression analysis, and proteomics is needed to identify more representative indicators.

## 6 Conclusion

Our study suggests that mitochondrial lymphocyte may be involved in the pathophysiology of schizophrenia and the weight changes induced by antipsychotic medications, and it may be associated with cognitive impairments, particularly in language function. Due to the cross-sectional nature of this study, it is necessary to further validate our findings by conducting larger sample sizes, longitudinal studies, and by comprehensively considering variations in drug use in future research.

## Data availability statement

The original contributions presented in the study are included in the article/**Supplementary Material**. Further inquiries can be directed to the corresponding authors.

## Ethics statement

The studies involving humans were approved by the Ethics Committee of Kangning Hospital in Wenzhou. The studies were conducted in accordance with the local legislation and institutional requirements. The participants provided their written informed consent to participate in this study. Written informed consent was obtained from the individual(s) for the publication of any potentially identifiable images or data included in this article.

## Author contributions

YZ: Formal analysis, Resources, Writing – original draft. WT: Data curation, Funding acquisition, Writing – original draft. BT: Methodology, Supervision, Writing – review & editing. KF: Investigation, Methodology, Writing – original draft. KZ: Software, Supervision, Writing – review & editing. XF: Conceptualization, Validation, Visualization, Writing – review & editing. HL: Project administration, Writing – review & editing.
