# Supplementary material for: Altered mitochondrial lymphocyte in overweight schizophrenia patients treated with atypical antipsychotics and its association with cognitive function
Source: Front Immunol. 2024 Jan 3;14:1325495. doi: 10.3389/fimmu.2023.1325495 (PMC10791827; doi:10.3389/fimmu.2023.1325495)
Supplement: Supplementary file 1 [file DataSheet_1.docx]

**Supplemenatry materials**

Table S1 Gender Differences in Clinical Characteristics of Schizophrenia Patients with and without Overweight

|  | Male | | t/X^2^ | p | Female | | t/X^2^ | p |
| --- | --- | --- | --- | --- | --- | --- | --- | --- |
|  | Overweight  (n=27) | Normal  (n=24) |  |  | Overweight  (n=26) | Normal  (n=20) |  |  |
| Age（year） | 39.22 ± 9.03 | 39.25 ± 9.53 | 0.11 | 0.992 | 41.42 ± 11.31 | 39.50 ± 9.67 | 0.608 | 0.546 |
| Age of onset (years) | 24.93 ± 9.09 | 24.08 ± 7.54 | -0.358 | 0.722 | 27.38 ± 13.49 | 26.15 ± 9.84 | 0.344 | 0.732 |
| Duration of medication (years) | 13.70 ± 7.94 | 14.63 ± 8.47 | 0.401 | 0.690 | 12.12 ± 7.35 | 13.20 ± 8.73 | -4.457 | 0.650 |
| DAD(mg) | 436.30 ± 216.35 | 455.57 ± 325.67 | 0.252 | 0.802 | 382.48 ± 287.91 | 389.75 ± 261.04 | 0.088 | 0.930 |
| CRP | 2.66 ± 3.36 | 1.39 ± 1.42 | -1.788 | 0.082 | 2.92 ± 2.79 | 4.02 ± 8.56 | -0.552 | 0.587 |
| Mito-CD4+/CD8+ | 1.64 ± 0.70 | 1.57 ± 0.74 | -0.325 | 0.746 | 1.84 ± 0.75 | 1.76 ± 0.54 | 0.436 | 0.665 |
| Mito-CD3+CD4+ | 711.19 ± 276.74 | 541.50 ± 185.96 | -2.536 | 0.014^*^ | 820.81 ± 357.71 | 638.65 ± 247.06 | 1.946 | 0.058 |
| Mito-CD3+CD4+ (%) | 38.63 ± 8.30 | 38.40 ± 6.13 | -0.011 | 0.911 | 41.07 ± 6.57 | 41.58 ± 7.44 | -0.244 | 0.808 |
| Mito-CD3+ | 1256.96 ± 449.56 | 1062.92 ± 415.81 | -1.594 | 0.117 | 1348.34 ± 415.63 | 1159.70 ± 425.81 | 1.510 | 0.138 |
| Mito-CD3+ (%) | 71.54 ± 7.49 | 71.49 ± 7.55 | -0.023 | 0.982 | 71.73 ± 5.58 | 70.85 ± 7.43 | 0.439 | 0.664 |
| Mito-CD3+CD8+ | 436.52 ± 175.26 | 408.63 ± 233.59 | -0.486 | 0.629 | 445.75 ± 188.35 | 425.60 ± 171.95 | 0.373 | 0.711 |
| Mito-CD3+CD8+ (%) | 27.43 ± 7.76 | 26.77 ± 7.55 | -0.288 | 0.774 | 25.64 ± 6.97 | 25.37 ± 6.24 | 0.138 | 0.891 |
| RBANS |  |  |  |  |  |  |  |  |
| Immediate memory | 50.63 ± 9.14 | 51 ± 14.11 | 0.110 | 0.913 | 55.15 ± 16.36 | 55.70 ± 15.15 | -0.116 | 0.908 |
| Attention | 72.67 ± 14.63 | 72.50 ± 13.40 | -0.042 | 0.966 | 79.00 ± 15.37 | 72.50 ± 14.94 | 0.346 | 0.731 |
| Visuospatial | 72.30 ± 15.22 | 74.33 ± 16.12 | 0.464 | 0.645 | 75.23 ± 14.98 | 77.05 ± 16.12 | -0.409 | 0.685 |
| Delayed memory | 54.11 ± 11.38 | 58.96 ± 18.31 | 1.119 | 0.270 | 68.31 ± 18.95 | 65.10 ± 19.49 | 0.562 | 0.577 |
| Language | 69.04 ± 12.45 | 67.63 ± 13.86 | -0.383 | 0.703 | 67.54 ± 16.20 | 71.55 ± 13.55 | -0.892 | 0.377 |
| Total score | 56.48 ± 7.95 | 59.13 ± 10.35 | 1.029 | 0.308 | 61.92 ± 12.77 | 62.65 ± 12.62 | -0.192 | 0.848 |
| PANSS |  |  |  |  |  |  |  |  |
| Total score | 102.74 ± 11.97 | 104.63 ± 16.02 | 0.479 | 0.634 | 106.88 ± 11.28 | 110.55 ± 14.20 | -0.976 | 0.334 |
| Cognitive factor | 12.15 ± 1.79 | 12.21 ± 2.72 | 0.094 | 0.925 | 12.46 ± 2.27 | 13.10 ± 2.08 | -0.982 | 0.331 |
| Depressive factor | 8.00 ± 2.59 | 8.42 ± 2.80 | 0.553 | 0.583 | 9.35 ± 1.74 | 9.75 ± 1.68 | -0.791 | 0.433 |
| Positive symptoms | 14.33 ± 3.63 | 15.08 ± 4.71 | 0.641 | 0.524 | 14.46 ± 3.61 | 15.55 ± 3.93 | -0.975 | 0.335 |
| Negative symptoms | 22.70 ± 3.89 | 23.67 ± 4.07 | 0.863 | 0.392 | 23.73 ± 2.60 | 23.85 ± 5.21 | -0.102 | 0. 920 |
| Excitation | 12.30 ± 3.09 | 10.63 ± 3.13 | -1.917 | 0.061 | 11.73 ± 3.72 | 12.90 ± 2.86 | -1.165 | 0.250 |

Abbreviations: DAD =Daily antipsychotic medication dosage (chlorpromazine equivalents), CRP = C-reactive protein, Mito = Mitochondria, RBANS = the Repeatable Battery for the Assessment of Neuropsychological Status, SCWT = the Stroop Color-Word Test.

Note: *p＜0.05. Data were presented in Mean ± SD.

Table S2 Differences in Demographic and Mitochondrial Lymphocyte Count in Lymphocytes Between Patients and Controls

|  | Schizophrenia  (N=97) | | Healthy controls  (N = 100) | F/X^2^ | p |
| --- | --- | --- | --- | --- | --- |
|  | NW(N=44) | OW(N=53) |  |  |  |
|  |  |  |  | F | p |
| Age（year） | 40.30 ± 10.18 | 39.36 ± 9.49 | 40.23 ± 6.82 | 0.191 | 0.826 |
| Mito-CD4+/CD8+ | 1.74 ± 0.73 | 1.66 ± 0.65 | 1.66 ± 0.72 | 0.239 | 0.788 |
| Mito-CD3+CD4+ | 764.96 ± 320.74 | 585.66 ± 218.78 | 843.13 ± 451.59 | 7.120 | 0.001^**^ |
| Mito-CD3+CD4+ (%) | 39.83 ± 7.53 | 39.84 ± 6.86 | 37.92 ± 7.14 | 1.743 | 0.178 |
| Mito-CD3+ | 1301.79 ± 431.55 | 1106.91 ± 418.30 | 1519.00 ± 743.50 | 7.432 | 0.001^**^ |
| Mito-CD3+ (%) | 71.63 ± 6.56 | 71.20 ± 7.42 | 68.84 ± 7.93 | 3.004 | 0.052 |
| Mito-CD3+CD8+ | 441.05 ± 180.10 | 416.34 ± 205.73 | 557.25 ± 307.96 | 6.123 | 0.003^**^ |
| Mito-CD3+CD8+ (%) | 26.55 ± 7.37 | 26.13 ± 7.64 | 25.51 ± 7.57 | 0.357 | 0.700 |
|  |  |  |  | X^2^ | p |
| Sex（male/female） | 27/26 | 24/20 | 61/39 | 1.551 | 0.473 |

Abbreviations: Mito = Mitochondria.

Note: *p＜0.05, **p＜0.01. Data were presented in Mean ± SD.
